# Behavioral determinants of physical activity across the life course: a “DEterminants of DIet and Physical ACtivity” (DEDIPAC) umbrella systematic literature review

**DOI:** 10.1186/s12966-017-0510-2

**Published:** 2017-05-02

**Authors:** Giancarlo Condello, Anna Puggina, Katina Aleksovska, Christoph Buck, Con Burns, Greet Cardon, Angela Carlin, Chantal Simon, Donatella Ciarapica, Tara Coppinger, Cristina Cortis, Sara D’Haese, Marieke De Craemer, Andrea Di Blasio, Sylvia Hansen, Licia Iacoviello, Johann Issartel, Pascal Izzicupo, Lina Jaeschke, Martina Kanning, Aileen Kennedy, Fiona Chun Man Ling, Agnes Luzak, Giorgio Napolitano, Julie-Anne Nazare, Camille Perchoux, Caterina Pesce, Tobias Pischon, Angela Polito, Alessandra Sannella, Holger Schulz, Rhoda Sohun, Astrid Steinbrecher, Wolfgang Schlicht, Walter Ricciardi, Ciaran MacDonncha, Laura Capranica, Stefania Boccia, on behalf of the DEDIPAC consortium

**Affiliations:** 1 Department of Movement, Human and Health Sciences, University of Rome Foro Italico, P.za Lauro de Bosis, 15, 00135 Rome, Italy; 2 Section of Hygiene - Institute of Public Health, Università Cattolica del Sacro Cuore, Rome, Italy; 3 Leibniz Institute for Prevention Research and Epidemiology - BIPS, Bremen, Germany; 4 Department of Sport, Leisure and Childhood Studies, Cork Institute of Technology, Cork, Ireland; 5 Department of Movement and Sports Sciences, Ghent University, Ghent, Belgium; 6 Department of Physical Education and Sport Sciences, University of Limerick, Limerick, Ireland; 7 Univ-Lyon, CarMeN laboratory, Inserm U1060, INRA U1397, Centre de recherché en Nutrition Humaine, Université Claude Bernard Lyon 1, INSA Lyon, Charles Merieux Medical School, Fr-69600 Oullins, France; 8 Council for Agricultural Research and Economics -Research Centre for Food and Nutrition, Rome, Italy; 9 Department of Human Sciences, Society, and Health, University of Cassino and Lazio Meridionale, Cassino, Italy; 10 Department of Medicine and Aging Sciences, ‘G. d’Annunzio’ University of Chieti-Pescara, Chieti, Italy; 11 Department for Sport and Exercise Sciences, University of Stuttgart, Stuttgart, Germany; 12 Department of Epidemiology and Prevention, IRCCS Istituto Neurologico Mediterraneo: NEUROMED, Pozzilli, Italy; 13 School of Health and Human Performance, Multisensory Motor Learning Lab, Dublin City University, Dublin, Ireland; 14 Molecular Epidemiology Group, Max Delbruck Center for Molecular Medicine in the Helmholtz Association (MDC), Berlin, Germany; 15 Centre for Preventive Medicine, School of Health and Human Performance, Dublin City University, Dublin, Ireland; 16 Institute of Sport, Exercise & Active Living, Victoria University, Melbourne, Australia; 17 Institute of Epidemiology I, Helmholtz Zentrum München, German Research Center for Environmental Health, Neuherberg, Germany; 18 Section of Hygiene - Institute of Public Health, Università Cattolica del Sacro Cuore, Fondazione Policlinico Universitario “Agostino Gemelli”, Rome, Italy; 19 Italian National Institute of Health, (Istituto Superiore di Sanita - ISS), Rome, Italy

**Keywords:** Physical activity, Behavioral determinants, Umbrella systematic literature review, Health promotion

## Abstract

**Background:**

Low levels of physical activity (PA) are a global concern and increasing PA engagement is becoming a priority in current public health policies. Despite the large number of studies and reviews available, the evidence regarding the behavioral determinants of PA is still inconclusive. Thus, the aim of this umbrella systematic literature review (SLR) was to summarize the evidence on the behavioral determinants of PA across the life course.

**Methods:**

A systematic online search was conducted on MEDLINE, ISI Web of Science, Scopus, and SPORTDiscus databases. The search was limited to studies published in English from January, 2004 to April, 2016. SLRs and meta-analyses (MAs) of observational studies that investigated the behavioral determinants of PA were considered eligible. The extracted data were assessed based on the importance of the determinants, the strength of evidence, and the methodological quality. The full protocol is available from PROSPERO (PROSPERO 2014:CRD42015010616).

**Results:**

Seventeen reviews on 35 behavioral determinants of PA were eligible for this umbrella SLR. Regardless of age, the most investigated determinants were those related with ‘screen use’ and ‘smoking’. For youth, probable positive evidence emerged for ‘previous PA’ and ‘independent mobility and active transport’ among children and adolescents. For the adult population, ‘transition to university’ and ‘pregnancy/having a child’ showed probable negative associations.

**Conclusions:**

Although the majority of the evidence was limited and most of the determinants were not associated with PA, this umbrella SLR provided a comprehensive overview of the associations between behavioral determinants and PA. Youth should be physically active in the early years and increase active transportation to/from school, independent mobility, and ‘free-range activities’ without adult supervision, whilst adult PA behaviors are mostly influenced by the life events. Finally, more research is needed that incorporates prospective study designs, standardized definitions of PA, objective measurement methods of PA assessment, and the use of interactionist and mediational approaches for the evaluation of different behavioral determinants influencing PA behaviors.

## Background

Physical activity (PA) is widely recognized, together with appropriate nutritional and mental status, to be an important component of healthy lifestyle [1]. Since the benefits of being physically active for the prevention and the management of the non-communicable diseases are well known [2], the European Union (EU) is strongly engaged in promoting health-enhancing physical activity [3] for all individuals independently from age and social status. However, in Europe, a large proportion of children, adolescents, adults, and older adults still adopts sedentary lifestyles [4] and, consequently, are more at risk of non-communicable diseases.

Several ecological models for the exploration of the lifestyle choice have been proposed [5–8], including individual (e.g., biological, psychological, and behavioral aspects), interpersonal (e.g., relationships with parents, relatives, peers, and socio-cultural networks), environmental (e.g., access/availability of tools/services, and proximal/distal built/natural surroundings), and policy (e.g., organizational and governmental aspects) dimensions. Within those dimensions, positive, negative, inconclusive, or no associations might exist between several determinants and PA. However, both determinants and PA present a great diversity in research designs, measurement approaches, populations studied, types of measurement, terminologies, which still make difficult to draw a comprehensive understanding. In general, the term ‘determinant’ is used to address causal variables also including correlates (i.e., multiple variables intervening in cause-effect relationships), whilst mediators (i.e., variables influencing a cause-effect relationship between variables), moderators (i.e., variables effecting the strength of a relationship between variables), and/or confounders (i.e., variables associated with the outcome that distort the observed relationships) are considered different variables [9, 10]. Furthermore, a lack of commonality exists in the PA terminology applied in the studies and different forms of PA are considered, ranging from unstructured daily activities, occupational PA, leisure time PA to structured PA (e.g., exercise, grassroots sports, and competitive sports) and considering the most relevant parameters of PA, such as frequency (e.g., daily, weekly, monthly), duration (e.g., total time of activity, rest intervals), and intensity (e.g., low, moderate, moderate-vigorous, vigorous, maximal efforts).

Within the individual dimension, the determinants related to behaviour are widely investigated. Commonly, behavioral determinants have been already defined as “those that can be eliminated or reduces through lifestyle or behavioral changes” [11]. Thus, among all ages, investigations into the behavioral determinants considered those related with major life changing events (e.g., pregnancy and retirement) [12], habits (e.g., smoking and alcohol consumption) [13], sedentary behaviors (e.g., TV viewing and video/computer game use) [14], and available mobility (e.g., active school transport and independent mobility) [15, 16]. They have been studied in a number of primary epidemiological studies, systematic literature reviews (SLRs), and meta-analyses (MAs). However, the diversity in research designs, theoretical and measurement approaches, population groups, determinants investigated, and PA outcomes, across the literature, makes it difficult to understand the evidence and to draw appropriate conclusions on the importance of these determinants in influencing PA behaviors.

Recently, the European Commission endorsed a Joint Programming Initiative to increase research capacity across Member States to engage in a common research agenda on a Healthy Diet for a Healthy Life [17] and the DEterminants of DIet and Physical ACtivity-Knowledge Hub (DEDIPAC-KH) project was created [18]. In order to expand knowledge and to develop new insights and solutions in PA, the Thematic Area 2 of the DEDIPAC-KH project organized and carried out an umbrella systematic literature review (SLR) [19] on all the possible determinants of PA. Overall, seven categories of determinants of PA have been identified: biological, psychological, behavioral, physical, socio-cultural, socio-economic, and policy determinants. Due to the amount of researches available, the DEDIPAC-KH Management Team decided to organize the findings in seven separated umbrella SLRs, each focused on a single category. The DEDIPAC-KH Management Team is conscious that splitting the categories may cause a lost in the analysis of the interactions between those categories of determinants which may share commonalities. However, this strategy was considered necessary for a clear dissemination of insights on the determinants of PA.

The aim of this umbrella SLR was to provide a systematic overview of studies investigating behavioral determinants of PA across the life course by evaluating existing SLRs and MAs. The summary also captures the different study designs and definitions applied for behavioral determinants and PA. Additionally, overall results of the retrieved SLRs and MAs have been evaluated in terms of the importance of the determinants, the strength of the evidence, and the methodological quality.

## Materials

The manuscript has been drafted following the PRISMA checklist [20]. A common protocol of the seven umbrella SLRs (biological, psychological, behavioral, physical, socio-cultural, socio-economic, and policy) was registered and is available on PROSPERO (Record ID: *CRD42015010616*), the international prospective register of systematic reviews [21]. Review title, timescale, team details, methods, and general information were all recorded in the PROSPERO register prior completing data extraction.

## Search strategy and eligibility criteria

**Fig. 1:**
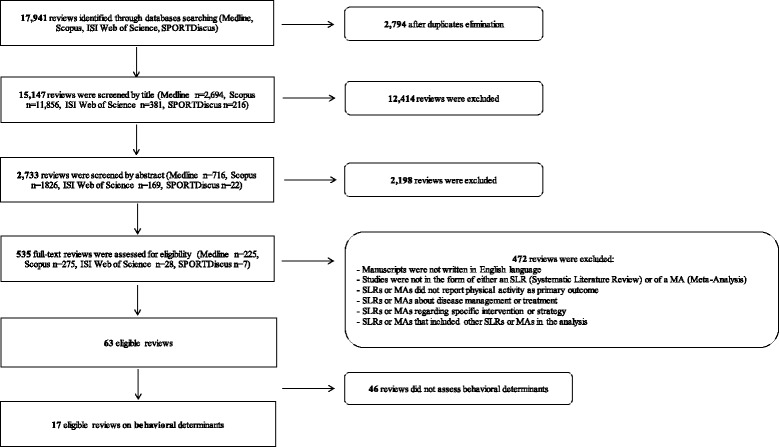
Flowchart of the literature research by database

The present study applied the same search strategy as was used for the other umbrella SLRs of the DEDIPAC-KH. SLRs and MAs investigating the determinants of PA across the life course were systematically searched on MEDLINE, ISI Web of Science, Scopus, and SPORTDiscus. The search was limited to SLRs and MAs published in English, between January 2004 and April 2016. To avoid duplications of the earliest individual studies, reviews published before 2004 were not included. Table 1 shows the MEDLINE search strategy, and Fig. 1 summarizes the process of the literature research, common to the subsequent 7 umbrella SLRs. Thus, Table 1 provides the overall list of searched terms, whilst Fig. 1 shows the count of the overall excluded/included reviews, related and not-related with behavioral determinants. SLRs or MAs of observational primary studies on the association between any determinant and PA, exercise (Ex) or sport as the main outcome, were included in the seven umbrella SLRs. The following studies were excluded: i) SLRs and MAs of intervention studies; ii) SLRs and MAs that focused on specific population groups (e.g., chronic diseases); and iii) umbrella SLR’s on the same topic (e.g., reviews of SLRs or MAs of epidemiological studies on variables in association with PA). In line with Peel and colleagues [10], behavioral determinants were defined as “those that can be eliminated or reduced through lifestyle or behavioral changes”.

**Table 1:** Search strategy: key words used for the literature research

Set	Search terms
#1	“physical activit*” OR “physical exercise*” OR sport OR “motor activit*” OR “locomotor activit*” OR athletic* OR fitness OR “physical movement*” OR “physical performance*” OR “aerobic exercise*” OR “physical effort*” OR “physical exertion*”
#2	determinant OR determinants OR correlate OR correlates OR mediator OR mediators OR moderator OR moderators OR contributor OR contributors OR factor OR factors OR association OR modifier OR modifiers OR confounder OR confounders OR pattern OR patterns OR predictor*
#3	demographic* OR motivation OR cognition OR emotion* OR attitude* OR “self-perception” OR “self-confidence” OR “self-efficacy” OR competence OR reward* OR success* OR challenge* OR knowledge OR belief* OR “personal trait*” OR “body image” OR satisfaction OR “time availability” OR “perceived environment” OR family OR peer* OR school* OR leader* OR coach* OR group* OR “climate” OR network* OR employment OR retirement OR “educational level” OR SES OR “socioeconomic status” OR “local identity” OR “national identity” OR value* OR tradition* OR “social expectation*” OR “social trend*” OR “social barriere*” OR “availability of tool*” OR “availability of service*” OR “access to tool*” OR “access to service*” OR neighborhood OR “community route*” OR “school environment” OR “work environment” OR architecture OR urbanization OR transport OR traffic OR “facilit* in public space*” OR advertisement OR “availability of sport club*” OR “availability of fitness center*” OR advocacy OR lobbying OR “corporate social responsibility” OR “physical activity promotion initiative*” OR legislation OR health OR education OR tourism OR environment OR “urban planning” OR transport* OR sport OR sports OR culture OR dance OR theater OR “gender mainstreaming” OR “social inclusion” OR “fiscal measure*” OR program* OR plan OR plans OR communication OR media OR guideline*
#4	“systematic literature review” OR “meta-analysis”

## Selection process

Relevant articles were independently screened and assessed by two reviewers belonging to the DEDIPAC-KH, who screened the titles, the abstracts, and the full texts. Before the final study inclusion or exclusion, a common decision was reached for each study. Any uncertainty and disagreement was resolved by consulting three further authors of the DEDIPAC-KH (BS, CL, PA).

## Data extraction

For each included review, data was extracted on a predefined data extraction form, developed by the DEDIPAC-KH and checked by two authors (AK, PA). In reporting data, authors agreed to use the terms “reviews” as those SLRs and MAs found eligible for the umbrella SLR, and ‘primary studies’ as those studies included in the eligible SLRs and MAs. Moreover, authors agreed to consider all of the terminologies and forms of PA, including unstructured (i.e., PA linked with daily life) and structured (i.e., exercise and sports) independently from their frequency, duration, and intensity.

The following information was extracted from each included review: year of publication, type of review (either SLR or MA), number of eligible primary studies included in the behavioral umbrella SLR over the total number of primary studies included in the review, continent/s of the included primary studies, primary study design, overall sample size, age range or mean age, gender proportion, year of publication range of included primary studies, outcome details, type of determinant/s, aim of the review, overall results (qualitative or quantitative), overall recommendations, and limitations as provided by the review itself.

## Evaluation of importance of determinants and strength of the evidence

The results retrieved from the eligible primary studies included in the reviews were summarized combining two slightly modified grading scales, previously used by Sleddens et al. [22]. The first scale, grades the importance of the determinants, referring only to the consistency and direction of the associations among the reviews, or the individual primary studies. The second scale, grades the strength of evidence, referring to the study design used among individual primary studies. For its importance, a determinant scored a “--” if all reviews, without exception, reported a negative association between the determinant and the outcome and a “-” if the negative association was found in more than 75% of the reviews or of the original primary studies. The importance of the determinant was scored a “0” if the results were mixed, or more specifically, if the variable was found to be a determinant and/or reported an association (either positive or negative) in 25 to 75% of available reviews or of the primary studies of these reviews, but not in others. Furthermore, the importance of the determinant scored a “+” if a positive association was found in more than 75% of the reviews or of the included primary studies and a “++” if a positive association was found in all reviews, without exception. Despite in the literature the codes “+” and “++” were used in presence of an association, independently from its direction (e.g., positive or negative) [22], in the present SLR these codes denote both the strength and positive direction of the association.

The strength of the evidence was described as ‘convincing’ (Ce) if it was based on a substantial (n > 10) number of longitudinal observational studies showing associations between the determinant and PA. The strength of the evidence was defined as ‘probable’ (Pe) if it was based on at least two cohort studies or five cross-control studies showing associations between the determinant and PA. Furthermore, the strength of the evidence was given as ‘limited suggestive evidence’ (Ls) if it was based mainly on findings from cross-sectional studies showing associations between the determinant and PA. Evidence was labelled as ‘limited, no conclusive evidence’ (Lns) if the study findings were suggestive but insufficient to provide an association between the determinant and PA [and if no longitudinal data available].

## Quality assessment

The methodological quality of the included reviews was assessed using a modified version of the AMSTAR Checklist [23]. After a consensus between the DEDIPAC-KH partners, the question (number 11) referring to the presence of any conflict of interest was modified to allow for any conflict of interest to be evaluated in any of the included reviews but not in the primary studies included in each review.

Two authors belonging to the DEDIPAC-KH independently evaluated the included reviews, using the same methodology of Sleddens et al. [22]. Any uncertainty and disagreement was resolved by consulting three further authors (SB, LC, AP). The eleven criteria were evaluated and scored with 1 when the criterion was applicable to the analyzed review or with 0 when the criterion was not fulfilled, not applicable to the analyzed review, or could not be answered based on the information provided by the review. As a consequence, the total quality score for each included review ranged from 0 to 11. The quality of the review was labelled as weak (score ranging from 0 to 3), moderate (score ranging from 4 to 7), or strong (score ranging from 8 to 11).

## Results

### SLRs and MAs selection process

As summarized in Fig. 1, the systematic literature search identified 17,941 reviews that were potentially relevant for inclusion in our umbrella SLR. After the removal of duplicates, 15,147 reviews remained for screening. After reading title and abstract, 12,414 and 2,198 reviews, respectively, were excluded because they did not meet the inclusion criteria. Thus, a total of 535 full-text reviews were assessed for eligibility. From these, another 472 reviews were removed because they did not meet the inclusion criteria. Thus, after the full-text reading phase, the number of reviews eligible for the umbrella review was 63. Of these, 46 reviews did not include behavioral determinants of PA. Therefore, the final number of reviews included in the present umbrella SLR on behavioral determinants of PA was 17 (15 SLRs and 2 MAs).

### Characteristics of the reviews and quality assessment

The characteristics of the 17 included reviews are summarized in Table 2. All the primary studies included in the individual SLRs or MAs referring to non-behavioral determinants and PA were not considered. Most of the reviews included primary studies from multiple continents, mostly from Europe, North America, and Australia. The predominant study design used among the primary studies was cross-sectional [12, 13, 15, 16, 24–33]. Nine reviews included prospective and cohort studies, either as the only eligible study design [34, 35] or as part of the included studies [12, 15, 16, 25, 26, 29, 32]. In six reviews, it was not possible to retrieve the total population sample size of the included primary studies because it was either not reported or not complete [26, 27, 29, 30, 32, 33]. In the remaining studies, the total population sample size ranged from 26 [12] to 82,918 [13].

**Table 2:** Characteristics of the included reviews

Author, Date (Type of review) [Ref]	Number of eligible studies included in the umbrella review/total number of studies included in the review	Continent/s of included studies	Study design of included studies	Total sample size of included study (Sample range)	Age range or mean (years) of eligible studies	Gender (female, % range) of eligible studies	Year range of included studies
Babakus WS, 2012 (SLR) [24]	6/38	Europe (*n* = 6)	In-depth interviews (*n* = 2) Focus group (*n* = 1) Semi-structured interviews (*n* = 1) N.A. (*n* = 2)	276 (30–109) N.A. (*n* = 2)	40–83 N.A. (*n* = 2)	39–53 N.A. (*n* = 2)	1980–2012
Barnett I, 2012 (SLR) [25]	19/19	North America (*n* = 11) Europe (*n* = 6) Australia (*n* = 2)	Cohort (*n* = 14) Cross-sectional (*n* = 5)	62,455 (51–11,469)	45–99	0–100	1985–2010
Craggs C, 2011 (SLR) [34]	13/46	North America (*n* = 11) Europe (*n* = 2)	Prospective (*n* = 13)	13,332 (40–3,878)	4–9 (*n* = 1) 10–13 (*n* = 8) 14–18 (*n* = 4)	N.A.	1986–2009
De Craemer M, 2012 (SLR) [26]	7/43	N.A.	Cross-sectional (*n* = 6) Longitudinal (*n* = 1)	N.A.	4–6	N.A.	2003–2010
Engberg E, 2012 (SLR) [12]	32/34	Europe (*n* = 7) North America (*n* = 22) Australia (*n* = 3)	Cross-sectional retrospective (*n* = 7) Prospective longitudinal (*n* = 25)	276,558 (26–80,944)	17–70	N.A.	1992–2012
Kaczynski AT, 2008 (SLR) [13]	50/50	North America (*n* = 23) Europe (*n* = 18) Australia (*n* = 3) Asia (*n* = 1) Multiple Continents (*n* = 1) N.A. (*n* = 4)	Mostly cross-sectional	381,807 (120–82,918)	6–89	N.A.	1970–2005
Koeneman MA, 2011 (SLR) [27]	2/34	Australia (*n* = 1) Asia (*n* = 1)	Observational (*n* = 2)	N.A.	40–80	N.A.	2007
Larouche R, 2014 (SLR) [15]	46/73	Europe (*n* = 25) North America (*n* = 9) South America (*n* = 1) Asia (*n* = 1) Australia (*n* = 9) Russia (*n* = 1)	Cross-sectional (*n* = 41) Prospective (*n* = 5)	66,489 (103–7,023)	5–17.9	53	2002–2012
Lee MC, 2008 (SLR) [28]	24/32	North America (*n* = 9) Europe (*n* = 10) Australia (*n* = 5)	Cross-sectional (*n* = 24)	33,756 (88–10,771)	5–18	N.A.	2002–2007
Marshall SJ, 2004 (MA) [14]	24/54	N.A.	N.A.	1443,235 (36–20,766)	0–18	N.A.	1990–2002
Pearson N, 2014 (MA) [29]	163/163	Multiple Continents	Prospective (*n* = 12) Cross-sectional (*n* = 147) Prospective and Cross-sectional (*n* = 4)	N.A.	0–18	N.A.	1987–2013
Ridgers ND, 2012 (SLR) [33]	3/53	Europe (*n* = 2) Australia (*n* = 1)	Cross-sectional (*n* = 2) N.A. (*n* = 1)	N.A.	5–18	N.A.	2006–2010
Schoeppe S, 2013 (SLR) [16]	42/52	Europe (*n* = 25) North America (*n* = 9) Asia (*n* = 1) Australia (*n* = 6) Russia (*n* = 1)	Cross-sectional (*n* = 39) Longitudinal (*n* = 3)	55,896 (103–6,085)	3–18	39–100	2002–2012
Stanley RM, 2012 (SLR) [30]	6/22	Europe (*n* = 1) North America (*n* = 4) Australia (*n* = 1)	Cross-sectional (*n* = 6)	N.A.	8–14	N.A.	1997–2010
Tzormpatzakis N, 2007 (SLR) [31]	3/36	Europe (*n* = 3)	Cross-sectional (*n* = 3)	4,213 (171–1,000)	15–89	50–54	2002–2004
Uijtdewillingen L, 2014 (SLR) [35]	11/30	Europe (*n* = 5) North America (*n* = 5) Asia (*n* = 1)	Prospective (*n* = 11)	11,259 (155–5,451)	4–18	51–100 (*n* = 10) N.A. (*n* = 1)	2005–2010
van der Horst K, 2007 (SLR) [32]	10/57	N.A.	Cross-sectional (*n* = 8) Prospective (*n* = 2)	N.A.	4–12 (*n* = 3) 13–18 (*n* = 7)	N.A.	1999–2004

Notes: MA: Meta-Analysis; SLR: Systematic Literature Review; N.A.: Not Applicable

Eleven reviews referred to primary studies that included young people only. Among these, preschool children aged between 4 and 6 years old were assessed in one review [26], whilst children and adolescents (8–18 years) were included in ten of the reviews [14–16, 28–30, 32–35]. Three reviews considered adults older than 40 years [24, 25, 27], while three other reviews considered the population as a whole [12, 13, 31]. Finally, the percentage of the female participants, when reported, ranged from 0 [25] to 100% [16, 25, 35], though that data was absent in the majority of the studies [12–14, 26–30, 32–34].

### Measurements of PA

From the 17 reviews included, 461 primary studies were found eligible. Among these, 218 studies from 15 reviews used non-objective measurement methods of PA assessment (e.g., self-report, parental report, direct PA observation) [12, 13, 15, 16, 24, 25, 27–35]. Objective measurements of PA, either assessed by accelerometer or pedometer, were used in 201 of the eligible primary studies and were included in nine of the included reviews [15, 16, 28–30, 32–35]. Eleven primary studies included in four reviews combined objective with non-objective measures of PA [15, 28, 29, 34]. Finally, 31 primary studies from two reviews did not report the exact number of the studies that used objective and non-objective measures [14, 26].

As reported in Table 3, the majority of the included reviews evaluated overall PA as an outcome (*n* = 13) [12–16, 24–26, 28, 29, 32, 34, 35]. One review also measured moderate-to-vigorous physical activity (MVPA) and active transport [26] next to overall PA. Two reviews measured overall PA/Ex [27, 31], one review measured time-specific PA (i.e., school break time PA and after-school PA) [30] and one review measured recess PA [33].

**Table 3:** Results of the included reviews

Author, Date (Type of review) [Ref]	Outcome(s)	Determinant(s)	Review aim	Overall qualitative results of the review	Overall quantitative results of the review	Overall limitations of the study	Overall Recommendations
Babakus WS, 2012 (SLR) [24]	Overall PA	Language difficulties; lack of time	To assess what is known about the levels of PA and sedentary time and to contextualize these behaviors among South Asian women with an immigrant background.	South Asian women are less active than the other ethnic groups as well as compared to South Asian males; knowledge of PA and its benefits was found to be lacking among south Asian.	N.A.	No standardized method for quality evaluation; lack of details from some of the included papers; measurement and definition of PA varied widely; publication and researcher bias possibility; significant heterogeneity among studies.	More research should be dedicated to standardize objective PA measurement and to understand how to utilize the resources of the individuals and communities to increase PA levels and overall health of South Asian women; future research is needed to assess levels of sedentary time and contextualize sedentary behaviors.
Barnett I, 2012 (SLR)[25]	Overall PA	Work after retirement from main occupation; retirement from strenuous occupation; being retired for <5 years; lifelong participation in PA; being married	To examine changes and predictors of changes in PA across the transition to retirement; whether these changes vary by SES; what is known about predictors of changes in PA across the retirement transition.	Exercise and leisure-time PA increased after the transition to retirement, whereas the findings regarding changes in total PA were inconclusive; men increase their PA more than women; lower SES is associated with a decrease and high SES with an increase in PA. Evidence on other predictors was scarce, often inconsistent, and methodologically weak.	N.A.	Evidence on predictors of change was scarce and methodologically weak; no language or country restrictions; published peer-reviewed journal articles as well as gray research literature were included; multidisciplinary approach contributed to the heterogeneity of the results and to the unfeasibility of meta-analysis.	Further studies should include other measures of SES, appropriate and valid PA measures, apply clear and relevant defınitions of retirement, and study predictors of PA change across the transition to retirement; qualitative studies and longitudinal studies with longer follow-up are needed.
Craggs C, 2011 (SLR) [34]	Overall PA	Vigorous PA; participation in sport teams outside school; previous PA; alcohol consumption; dietary habits; smoking status; sedentary behavior	To collate the current evidence base, highlight research trends and limitations in physical activity determinants research, and synthesize the existing evidence.	Inconclusive associations were reported for large proportion of the determinants examined; girls consistently reported larger declines in PA than boys in younger children; parental marital status was consistently shown not to be associated with change in activity; higher levels of self-efficacy were associated with smaller declines compared to lower levels of self efficacy in older children and adolescents.	N.A.	Possibility of publication bias (included published studies only); heterogeneity in study samples, exposure and outcome measures included in this review; some studies draw data from the same cohorts; semi-quantitative reporting used in the review that limits the classification of the associations.	Further research should include objective measures of PA and use previously validated questionnaires to assess the investigated determinants; more high quality research is needed in all age groups, especially in younger children; investigation into determinants of change should take into account of specifıc physical intensities such as minutes spent in moderate or vigorous physical activity.
De Craemer M, 2012 (SLR) [26]	Overall PA, MVPA, active transport	Participation in organized sports; television viewing/sedentary; enjoy television viewing; child has no energy to use active transport	To systematically review the correlates of PA, sedentary and eating behavior in preschool children 4-6 years old.	Attending a rural preschool was positively associated with physical activity; gender, age and socioeconomic status were not associated with physical activity, while an indeterminate result was found for ethnicity; gender and ethnicity were not associated with sedentary behavior and indeterminate results were found for age and socioeconomic status; preschoolers were more physically active as well as sedentary on weekdays; watching television was associated with a higher consumption of snacks and sweet beverages.	N.A.	Some limitations regarding the coding of the association of the variables; new categories for each behavior were made.	Future research should focus on identifying the common correlates of physical activity, sedentary behavior and eating behavior in preschool-aged children so that better tailored interventions could be developed. Furthermore, more longitudinal studies could contribute in drawing stronger conclusions on determinants of these EBRBs.
Engberg E, 2012 (SLR) [12]	Overall PA	Transition to university; change in employment status; marital transitions and changes in relationships; pregnancy/having a child; experiencing harassment at work, violence or disaster; moving into an institution	To examine literature concerning the effects of life events on changes in PA.	Most of the studies reviewed showed statistically significant changes in PA associated with certain life events; transition to university, having a child, remarriage and mass urban disaster were associated with decreased PA levels, while retirement was associated with increased PA; experiencing multiple simultaneous life events were associated with decreased PA in men and women; PA is often used as part of a rehabilitation programme for diseases.	N.A.	Self-reported PA data are likely to be somewhat limited; another limitation is the possible cross contamination of responses when PA levels before and after a life event were assessed at the same time; the generalization of study findings to lower socioeconomic status and ethnic minority populations, as well as to other countries, may be limited because PA and life events vary; limitations of the studies include that PA data were not always reported in detail; some studies did not analyze, statistically, the magnitude of change in PA; another important limitation is that life events tend to overlap.	Future studies should examine gender differences in the effects of life events and use validated methods in assessing leisure PA. Longitudinal cohort studies and clinical trials from different countries and cultures are needed. Studies with longer follow-ups are necessary to examine how long the effects of life events on PA persist.
Kaczynski AT, 2008 (SLR) [13]	Overall PA	Smoking	To better understand the co-occurrence of smoking and physical inactivity in both adults and youth.	In approximately 61% of the studies of adult populations, the association between smoking and PA was negative, less pronounced for youth and adolescents, and among males than females.	N.A.	Some authors defined a smoker as someone who smoked at least once in the past 30 days whereas other studies used the criterion of at least one cigarette per day over the past month. Further complicating interpretations is the fact that controlling for or including different variables changes the impact of the "predictor" variables on the "outcome" measures. Almost all of the aforementioned studies were cross-sectional, and so inferring causality or the direction of relationships is near impossible.	Researchers hoping to improve understanding of the joint relation between PA and smoking need to collect data at both the individual and organization (i.e., environment) levels. Understanding the mechanisms by which depression, lung function, school setting, or other factors mediate or moderate the smoking and PA relationship requires measurement of these variables at multiple (preferably three or more) points in time. By expanding the array of investigative methods employed, researchers will be better equipped to understand linkages between smoking and PA and to design effective interventions.
Koeneman MA, 2011 (SLR) [27]	Overall PA, Overall Ex, Overall PA/Ex	Baseline activity level; smoking	To systematically review determinants of PA and exercise among healthy older adults.	Insufficient evidence for most associations between possible determinants and PA or Ex.	N.A.	There may be possibility of publication bias; a wide age range is applied that might have masked some of the differences between subsamples inside that population; they excluded some specific subsamples of the older population; overall low quality of the studies included	The determinants of PA need further study that include the use of objective measures of PA and exercise and valid and reliable measures of determinants.
Larouche R, 2014 (SLR) [15]	Overall PA	Active school transport	To systematically assess the quality of evidence with respect to the relationship between active school transport and daily PA, cardiovascular fitness, and body composition.	Overall, 81.6% of the studies showed positive associations between active school transport and PA levels with moderate quality of evidence.	N.A.	Meta-analyses were precluded due to the wide heterogeneity in study methodologies and analyses. Further, the funnel plot and related statistical methods for detecting publication bias could not be used because a consistent measure of effect across studies was not available.	Future active school transport studies should analyze walking and cycling separately. Future studies could evaluate the impact of existing programs that promote active school transport on PA levels and health related outcomes. Future studies should consider assessing active school transport as a continuous variable (i.e., frequency × distance).
Lee MC, 2008 (SLR) [28]	Overall PA	Active commuting to school	This article presents a systematic review of the literature on active commuting (walking or bicycling) and the outcomes of physical activity and weight/obesity in school-age children.	In multiple studies in a number of countries, a significant association between physical activity and walking or cycling to school has been noted across many age groups of school children. The strength of the association is mixed and a summary estimate is not possible because of the heterogeneity in study design. Regardless of this association, it remains unclear as to whether the commute trip itself is a significant component of total physical activity.	N.A.	The definition of active commuting and the potential for misclassification bias; the methodology of measuring physical activity; the majority of cross-sectional studies.	Future research should focus on measuring the impact of interventions that succeed in changing commuting behavior and quantifying the contribution of the commute to children’s daily activity.
Marshall SJ, 2004 (MA) [14]	Overall PA	TV viewing; video/computer game use	To examine the evidence that sedentary behaviors displace physical activity.	Possible mechanisms lack supporting evidence and claims that TV viewing, playing video games or using computers displace physical activity receive very limited empirical support.	−0.096 (95% CI = -0.080,-0.112) -0.104 (95% CI = -0.080,-0.128)	Only studies written in English.	More experimental research is needed to replicate these findings and explore possible mechanisms.
Pearson N, 2014 (MA) [29]	Overall PA	Total sedentary behavior; computer; homework; internet; reading; screen time; television; video games; composite sedentary behavior	The aim of the current study was to systematically review and meta-analysis peer-reviewed research describing the association between sedentary behavior and PA in children and adolescents.	A significant, but small, negative association was found between sedentary behaviors and physical activity in children and adolescents; in moderator analyses, studies that recruited smaller samples, employed objective methods of measurement or were assessed to be of higher methodological quality reported stronger associations, although the magnitude of effect remained small or small to moderate; small inverse associations were observed between specific sedentary behaviours, including Internet use, screen time and TV viewing, and physical activity.	−0.108 (95% CI = -0.128,-0.087) -0.018 (95% CI = -0.038,0.001) 0.014 (95% CI = -0.043,0.095) -0.051 (95% CI = -0.097,-0.006) -0.009 (95% CI = -0.039,0.021) -0.080 (95% CI = -0.101,-0.060) -0.064 (95% CI = -0.084,-0.045) -0.002 (95% CI = -0.043,0.040) -0.265 (95% CI = -0.364,-0.165)	Dichotomization of a continuous construct; searches were confined to studies published in peer-reviewed journals and those written in English; evaluation of associations between sedentary behavior and all PA outcomes combined, instead of different PA domains.	Further research is required to clarify the relative and interacting impact of PA and sedentary behavior on weight status in this population.
Ridgers ND, 2012 (SLR) [33]	Recess PA	Sport activities; physical conflict frequency; day-to-day variability; play ball games; sedentary activities	To examine the correlates of children’s and adolescent’s PA during school recess periods.	Higher perceived encouragement from parents, peers, and the school as a whole was associated with higher self-reported physical activity levels during recess periods, particularly in adolescents; overall facility provision (i.e., sum of facilities available) was positively associated with physical activity; a positive association was found between unfıxed equipment and recess physical activity in children; boys are more physically active during recess.	N.A.	The majority are small-sized and cross-sectional studies; meta-analysis is difficult to obtain given the limited number of studies and the lack of consistency in correlates assessed; a range of physical activity measures have been used; different cut-points and observation systems may have influenced the strength of associations observed.	Further research should investigate correlates of children’s and adolescents’ recess physical activity for variables that were not investigated frequently but indicated positive associations with physical activity; further research using objective measures is needed to determine adolescents’ recess physical activity levels; future research should examine whether increasing access to school facilities during recess periods increases physical activity levels in children and adolescents; further research is needed to determine whether specifıc types of equipment, or the overall availability of unfıxed equipment, are associated with higher levels of physical activity; future research should examine the correlates of boys and girls physical activity separately; More research is needed concerning correlates of PA in recess period, particularly in adolescents.
Schoeppe S, 2013 (SLR) [16]	Overall PA	Independent mobility without adult supervision (Active travel to and/or from school and active travel to leisure time related places); independent mobility without adult supervision (outdoor play); active travel with or without adult supervision (Active travel to and/or from school); active travel with or without adult supervision (Active travel to leisure time related places)	To synthesize the evidence for associations of children’s independent mobility and active travel to various destinations with physical activity, sedentary behavior and weight status.	Children’s active travel to and/or from school was consistently positively associated with physical activity; positive relationships were found between active travel to leisure-related destinations and physical activity; travel to various places including school, shops, cinemas and friend’s homes were positive associated with moderate-to-vigorous and overall physical activity; children were more likely to increase physical activity levels when their outdoor play occurred independently; associations between active school travel and weight status were inconsistent across the studies.	N.A	Few studies measuring active travel to places other than school; use of different thresholds for defining objectively measured sedentary behavior in children; the measurement of sedentary activities in children still lacks standardization and validation; Social and physical environments conducive to children’s independent mobility and active travel were not explored but certainly play a role as correlates of children’s physical activity, sedentary behavior and weight status.	Future studies should seek to investigate associations of independent mobility and active travel with light intensity physical activity; children’s daily active travel to various destinations beyond the school setting requires further attention, as well as investigating diverse sedentary behaviors beyond simply screen-based activities and defining appropriate thresholds for objectively measured sedentary behavior in children; the potential of independent mobility to prevent sedentary behavior and excessive weight should be explored in future observational studies; studies should measure independent mobility and active travel more thoroughly by considering frequency and duration of independent mobility and active travel, as well as whether children are travelling alone or with peers, or to proximal versus distant destinations, and whether the observed associations differ by age and gender.
Stanley RM, 2012 (SLR) [30]	Time-specific PA (i.e., school break time PA and after-school PA)	TV viewing/playing video games; use of facilities; member of organized activities	To identify the correlates of childrens' PA (8-14years) occurring during the school break-time and after school periods.	Boys and younger children tend to be more active during break-time and after-school; BMI in females negatively associated with after-school PA, age was negatively associated in school-break and after school; family affluence, access to a gym, access to four or more PA programs and the condition of a playing field were all associated with school break time PA in one study; access to loose and fixed equipment, playground markings, size of and access to play space and the length of school break time were all positively associated with changes in school break time PA in intervention studies; in the after-school period, gender (with boys again more active), younger age, lower body mass index (for females), lower TV viewing/playing video games and greater access to facilities were associated with higher levels of after-school PA in two or more studies, while parent supervision was negatively associated with females’ after-school PA in one study.	N.A.	Small number of studies that vary in methodological aspects; possibility that some studies are missed during the search process; majority of cross-sectional studies; some studies stratified analyses by salient variables such as age, gender and intensity of PA, resulting in an over-representation of these studies in the review; the relatively narrow age range specified in the current review is a limitation.	Future studies using a context specific approach should identify and report specific facilities relevant to the context in question, which will contribute to a clearer understanding of context-specific PA; future studies should choose measurement tools with appropriate psychometric properties; Need of high quality evidence upon which PA promotion in young people can be tailored to specific settings and contexts.
Tzormpatzakis N, 2007 (SLR) [31]	Overall PA/Ex	Lack of time; time constraints; marital status	To evaluate the evidence from research relevant to participation in PA and exercise in Greece.	Participation in physical activity and exercise in Greece was generally low; men exercise more vigorously and more actively than women; the main reasons for participation were health, weight control, fitness, and stress management. Lack of leisure time was the main perceived barrier to increasing levels of physical activity and exercise; men seemed to exercise more frequently and more vigorously than women; single people were more likely to be physically active compared to those who were married or divorced/widowed; people living in rural areas were more likely to be physically active than those living in urban areas.	N.A.	None of the studies used objective measurements and also they used different self-reported estimates of PA; lack of appropriate use of the terms 'exercise' and 'PA'; none of the instruments were validated in Greece; seasonality effect, since seasonal variation exists in physical activity participation.	PA promotion should be organized in a systematic way; a clear definition of variables is needed; intervention studies should be employed in order to test the efficiency of policies, strategies and campaigns; longitudinal studies should be utilized in order to evaluate trends in physical activity participation and the long-term effects of campaigns; a clear pattern of who is considered physically active and inactive should be determined according to internationally established criteria in order to have more valid and reliable data; studies should not concentrate only on leisure time physical activities but on the total physical activity profile of each participant.
Uijtdewillingen L, 2014 (SLR) [35]	Overall PA	Past physical activity; screen time	To summarize and update the existing literature on determinants of PA and sedentary behavior in young people.	Moderate evidence for intention as a determinant of children’s physical activity, and age (i.e., older children were more active), ethnicity (i.e., being of African–American descent determined being less physically active) and planning as determinant of adolescent physical activity.	N.A.	Included studies assessed overall PA only; used two databases only; the selected language of publication was English only.	Future researches should be focused on determinants of child and adolescent sedentary behavior and on environmental determinants of physical activity in both children and adolescents, should use reliable and valid measures of both determinants as well as the actual behaviors and should conduct prospective studies of high methodological quality.
van der Horst K, 2007 (SLR) [32]	Overall PA	PE/school sports; watching TV; smoking; television/sedentary time	To summarize and update the literature on correlates of PA, insufficient PA, and sedentary behavior in young people.	The results from our review suggest that correlates of physical activity for children are gender, self-efficacy, parental physical activity for boys, and parental support. Correlates for adolescents' physical activity were gender, parental education, attitude, self-efficacy, goal orientation/motivation, physical education/school sports participation, family influences, and friend support. Gender, self-efficacy, and family/parental support were associated with physical activity, both in children and adolescents.	N.A.	Publication bias may be present; possibility of missed studies as a result of the search strategy; the main outcome was overall PA without other classifications; mostly cross-sectional studies included; because of the variability, it was not possible to assess the overall strength of the associations.	More prospective studies are needed and more research including children.

Notes: *MA* meta-analysis, *SLR* systematic literature review, *PA* physical activity, *PE* physical education, *MVPA* moderate-to-vigorous physical activity, *Ex* exercise, *SES* socio-economic status, *EBRB* energy balance-related behavior

### Categorization of the included determinants

During the preliminary phase, 52 behavioral determinants of PA were identified. Among those, similar determinants were identified to form a single determinant. For example, the determinants ‘*TV viewing*’, ‘*TV viewing/playing video games*’, ‘*television viewing/sedentary*’, ‘*enjoy television viewing*’, ‘*video/computer game use*’, ‘*computer*’, ‘*screen time*’, and ‘*video games*’ were merged into the determinant ‘screen use’. Differently, other similar determinants were identified to form a sub-group with a specific label. For example, the determinants ‘being *retired*’, ‘*change in employment status*’, and ‘*moving into an institution*’ were part of the sub-group ‘job-related’. Thus, a final consensus between authors had been achieved for this categorization and the final number of behavioral determinants was 35 (Table 4). Any uncertainty and disagreement was resolved by consulting three further authors of the DEDIPAC-KH (BS, CL, PA).

**Table 4:**
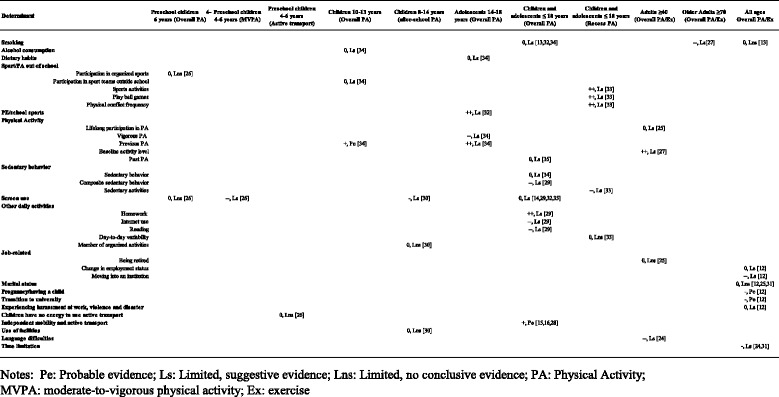
Summary of the results of the included reviews: the importance of a determinant and its strength of evidence

### Findings of the reviews

The findings of the included reviews on the associations between the behavioral determinants and PA, considering different age groups and different types of PA, are summarized in Table 4.

#### Preschool children

One review specifically assessed the behavioral correlates of PA in preschool children that were aged between 4 and 6 years old [26]. Among the correlates investigated, only those related with ‘screen use’ were negatively associated with MVPA in all the studies included in the review with a limited suggestive level of evidence (−−, Ls). Inconclusive findings were found regarding ‘participation in organized sports’ (0, Lns) and ‘screen use’ (0, Lns) with respect to overall PA and no association was found between ‘children have no energy to use active transport’ and active transport (0, Lns).

#### Children

Few determinants were investigated among children aged between 10 and 13 years old in respect to overall PA [34]. ‘Previous PA’ was the only determinant positively associated with overall PA in more than 75% of the studies included in the review with a probable level of evidence (+, Pe). Conversely, ‘participation in sport teams outside school’ (0, Ls) and ‘alcohol consumption’ (0, Ls) were not associated with overall PA in children. Considering after-school PA, one review investigated the behavioral correlates of PA in children between 8 and 14 years old [30]. Among the correlates investigated, only those related with ‘screen use’ were negative associated with after-school PA in more than 75% of the studies included in the review with a limited suggestive level of evidence (−, Ls). ‘Member of organized activities’ (0, Lns) was not associated with after-school PA, whilst for ‘use of facilities’ the evidence was limited and inconclusive (0, Lns).

#### Adolescents

Two reviews investigated the behavioral determinants [34] and correlates [32] of PA in adolescents aged between 14 and 18 years [32, 34]. ‘PE/school sports’ [32] and ‘previous PA’ [34] were both positively associated with overall PA in all the studies included in the review, without exception, both with a limited suggestive level of evidence (++, Ls). ‘Vigorous PA’ was negatively associated with overall PA in all the studies included in the review, without exception, with a limited suggestive level of evidence (−−, Ls) [34]. No association was found between ‘dietary habits’ and overall PA (0, Ls) [34].

#### Children and adolescents

Ten reviews examined the behavioral determinants and correlates of PA for children and adolescents (≤18 years old) in relation to overall PA [13–16, 28, 29, 32, 34, 35] and recess PA [33]. Independent mobility and active transport’ [15, 16, 28] was positively associated with overall PA in more than 75% of the studies included in the reviews, with a probable level of evidence (+, Pe). ‘Homework’ [29] was positively associated with overall PA in all the studies included in the review, without exception, with a limited suggestive level of evidence (++, Ls). In particular, a positive effect size (*r* = 0.014, 95% CI = −0.043, 0.095) emerged from the results of the MA [29]. Conversely, ‘Internet use’, ‘reading’, and ‘composite sedentary behavior’ were negatively associated with overall PA in all the studies included in the review, without exception, with a limited suggestive level of evidence (−−, Ls). The results of the MA [29] showed small to moderate negative effect sizes for the three determinants (*r* = −0.051, 95% CI = −0.097, −0.006; *r* = −0.009, 95% CI = −0.039, 0.021; *r* = −0.265, 95% CI = −0.364, −0.165, respectively). Limited evidence was found regarding ‘smoking’ (0, Ls) [13, 32, 34], ‘screen use’ (0, Ls) [14, 29, 32, 35], ‘sedentary behavior’ (0, Ls) [34], and ‘past physical activity’ (0, Ls) [35]. ‘Sports activities’, ‘playball games’, and ‘physical conflict frequency’ were positively associated with recess PA in all the studies included in the review, without exception, with a limited suggestive level of evidence (++, Ls) [33]. ‘Sedentary activities’ was negatively associated with recess PA in all the studies included in the review, without exception, with a limited suggestive level of evidence (−−, Ls) [33]. No association was found between ‘day-to-day variability’ and recess PA (0, Lns) [33].

#### Adults

Three reviews assessed the behavioral determinants of PA in adults older than 40 years [24, 25, 27]. ‘Baseline activity level’ was positively associated with overall PA/Ex in all the studies included in the review, without exception, with a limited suggestive level of evidence (++, Ls) [27]. Conversely, a negatively association was found between ‘language difficulties’ [24] and overall PA/Ex in all the studies included in the review, without exception, with a limited suggestive level of evidence (−−, Ls). For ‘being retired’ (0, Lns) and ‘lifelong participation in PA’ (0, Ls), the evidence was limited and inconclusive [25].

#### Older adults

For older adults (≥70 years), only one review [27] investigated the association between ‘smoking’ and overall PA/Ex suggesting negative associations in all the studies included in the review, without exception, with a limited suggestive level of evidence (−−, Ls).

#### All ages

Five reviews examined the behavioral determinants of PA across all ages [12, 13, 24, 25, 31]. A negative association was found between ‘moving into an institution’ and overall PA/Ex in all the studies included in the review, without exception, with a limited suggestive level of evidence (−−, Ls) [12]. ‘Pregnancy/having a child’ and ‘transition to university’ were negatively associated with overall PA/Ex in more than 75% of the studies included in the review with a probable level of evidence (−, Pe) [12], while ‘time limitation’ was negatively associated with overall PA/Ex in more than 75% of the studies included in the reviews with a limited suggestive level of evidence (−, Ls) [24, 31]. Finally, for ‘marital status’ (0, Lns) [12, 25, 31], ‘smoking’ (0, Lns) [13], ‘change in employment status’ (0, Ls), and ‘experiencing harassment at work, violence, and disaster’ (0, Ls) [12] the evidence was limited and inconclusive.

#### Evaluation of the quality of the SLRs and MAs

The results of the quality assessment using the AMSTAR checklist are reported in Table 5. Among the 17 included reviews, the majority were of moderate quality (*n* = 13) [12, 14–16, 24–28, 30, 33–35], three were weak [13, 31, 32], and only one was considered to be of strong quality [29]. Among those reviews of moderate quality, five were scored with 4 points [12, 26, 28, 30, 33], one with 5 points [16], four with 6 points [14, 15, 24, 34], and three with 7 points [25, 27, 35].

**Table 5:** Quality assessment of the included reviews using the AMSTAR Checklist

Author, Date (Type of review) [Ref]	Was an ‘a priori’ design provided?	Was there duplicate study selection and data extraction?	Was a comprehensive literature search performed?	Was the status of publication (i.e., grey literature) used as an inclusion criterion?	Was a list of studies (included and excluded) provided?	Were the characteristics of the included studies provided?	Was the scientific quality of the included studies assessed and documented?	Was the scientific quality of the included studies used appropriately in formulating conclusions?	Were the methods used to combine the findings of studies appropriate?	Was the likelihood of publication bias assessed?	Was the conflict of interest included?	Sum quality score^a^	Quality of the review^b^
Babakus WS, 2012 (SLR) [24]	No	Yes	Yes	Yes	No	Yes	Yes	No	Yes	No	No	6	Moderate
Barnett I, 2012 (SLR) [25]	No	Yes	Yes	Yes	No	Yes	Yes	Yes	Yes	C.A.	No	7	Moderate
Craggs C, 2011 (SLR) [34]	Yes	Yes	No	No	No	Yes	Yes	Yes	N.A.	No	Yes	6	Moderate
De Craemer M, 2012 (SLR) [26]	Yes	Yes	Yes	No	No	No	No	N.A.	N.A.	No	Yes	4	Moderate
Engberg E, 2012 (SLR) [12]	No	Yes	Yes	No	No	Yes	No	No	No	No	Yes	4	Moderate
Kaczynski AT, 2008 (SLR) [13]	No	No	C.A.	Yes	No	Yes	No	No	N.A.	No	Yes	3	Weak
Koeneman MA, 2011 (SLR) [27]	No	Yes	Yes	No	No	Yes	Yes	Yes	C.A.	Yes	Yes	7	Moderate
Larouche R, 2014 (SLR) [15]	Yes	Yes	Yes	No	No	Yes	Yes	Yes	No	No	No	6	Moderate
Lee MC, 2008 (SLR) [28]	Yes	No	Yes	No	No	Yes	No	No	No	No	Yes	4	Moderate
Marshall SJ, 2004 (MA) [14]	Yes	Yes	Yes	C.A.	No	No	Yes	Yes	Yes	No	No	6	Moderate
Pearson N, 2014 (MA) [29]	No	Yes	Yes	Yes	No	Yes	Yes	Yes	Yes	Yes	Yes	9	Strong
Ridgers ND, 2012 (SLR) [33]	Yes	C.A.	Yes	No	No	Yes	No	N.A.	N.A.	N.A.	Yes	4	Moderate
Schoeppe S, 2013 (SLR) [16]	Yes	No	Yes	No	No	Yes	Yes	Yes	N.A.	No	No	5	Moderate
Stanley RM, 2012 (SLR) [30]	No	Yes	No	No	No	No	Yes	Yes	N.A.	No	Yes	4	Moderate
Tzormpatzakis N, 2007 (SLR) [31]	No	C.A.	Yes	No	No	Yes	No	C.A.	N.A.	No	No	2	Weak
Uijtdewillingen L, 2014 (SLR) [35]	Yes	Yes	Yes	No	No	Yes	Yes	Yes	N.A.	N.A.	Yes	7	Moderate
van der Horst K, 2007 (SLR) [32]	No	Yes	Yes	No	No	Yes	No	N.A.	N.A.	No	No	3	Weak

Notes:

*C.A.* can’t answer, *N.A.* not applicable

^a^0 when the criteria was not applicable for the included review; 1 when the criteria was applicable for the included review

^b^Weak (score ranging from 0 to 3); Moderate (score ranging from 4 to 7); Strong (score ranging from 8 to 11)

Few reviews provided all the characteristics of the primary studies [12, 13, 15, 16, 25, 28, 31, 34, 35] and none of the reviews provided a full list of included and excluded studies.

## Discussion

This umbrella SLR aimed to provide a comprehensive and systematic overview of behavioral determinants of PA across the life course, evaluating importance, strength of the evidence, and methodological quality. Through a systematic evaluation of the existing SLRs and MAs, an overview of the associations between the considered behavioral determinants and PA is presented to promote effective health enhancing policies and identify gaps for future research strategies. To our knowledge, this is the first umbrella SLR that examined various potential behavioral determinants of PA across the life course.

Whether or not individuals choose to engage in PA behaviors is affected by a number of inter-dependent and multilevel factors. A relevant contribution of the DEDIPAC-KH project was the development of a dynamic framework of determinants of PA, which identified two main themes related to the person and to the society, respectively [36]. Behavioral determinants are part of the individual theme, together with the biological and psychological determinants, whereas the other theme comprises physical (e.g., environmental), socio-cultural, socio-economic, and policy determinants. Through the systematic analysis of scientific evidence, the present findings expand the knowledge and the understanding of behavioral determinants of PA to prepare the ground for a coherent approach towards PA behaviors research and interventions to favor active lifestyle choices.

Overall, for the majority of determinants, the evidence was limited and inconclusive or there was not an association with the different forms of PA. Furthermore, for several potential determinants it was not possible to ascertain definite conclusions on their association with PA. These findings urge researchers to consider new and innovative approaches when investigating factors influencing PA behaviors.

In relation to the investigated populations, more studies (*n* = 11) focused on youth (e.g., ≤18 years) with respect to those on individuals older than 18 years (*n* = 5). The reason for a greater focus on young populations might be due to the fact that being physically active at the youngest stages of age is crucial to maintain high levels of PA at the adulthood [32, 37], decreasing the risk factors for non-communicable diseases [2] and having a favorable effect on public health [37]. Nonetheless, the understanding of the relationships between behavioral determinants and PA is also crucial among older adults as they are more vulnerable and more likely to have decreasing level of PA and increasing levels of sedentary behaviors.

Among all the young population groups, 7 determinants were positively associated and 6 determinants were negatively associated with PA. A probable positive association was evident between ‘previous PA’ and overall PA in children, with the review showing a moderate quality [34], confirming the importance of PA in childhood. Moreover, a probable positive association was found in three reviews [15, 16, 28] between ‘independent mobility and active transport’ and overall PA in children and adolescents, with the reviews showing a moderate quality. Larouche and colleagues [15] argued that it is likely that an active traveler is also more inclined to be active throughout the day. However, other determinants (e.g., low socioeconomic status and lower income households), which were not considered in their review, may influence the relationship between ‘independent mobility and active transport’ and PA. Furthermore, potential biases due to classification, such as active school transport only once per week and direction of active transport (e.g., only to school and not from school) could also play a role. Other relevant aspects proposed by Schoeppe and colleagues [16] concern the positive associations of active travel to destination other than school, the health benefits for children of being involved in ‘free-range’ activities, like active travel or play outdoor without adult supervision, and the higher frequency of active travel from school to home rather than from home to school. Other determinants related to ‘sport/PA in and out of school’ [32, 33] and to ‘other daily activities’ [29] were positively associated with PA but with a limited suggestive level of evidence. The present findings are in line with the determinants related to school physical activity and youth sport participation (e.g., “PA Programs in School”, “Availability/Access/Proximity of PA Organized Sport Facilities/Tools”, “PA Education (at School/Work)/Knowledge of Effects of PA”, “Group Activities (Outdoor/Indoor)”, “Involvement in Organized Sport”, “Time Spent Outdoor/Playing Spaces”) highlighted by the European PA determinants (EU-PAD) framework as having a high priority for research [36]. To improve the strength of evidence, future research adopting longitudinal research designs is strongly needed. Indeed, motor skills in early childhood, school physical education, and youth sports are important aspects for a holistic development of children and adolescents, so an effective interaction between researchers and policy makers should be established to promote of health-enhancing lifestyles in early years, in line with the recommendations of the Expert Group on Health-Enhancing Physical Activity of the European Commission [38].

The determinants ‘composite sedentary behavior’, ‘internet use’, ‘reading’, ‘sedentary activities’, and ‘vigorous PA’ [26, 29, 33, 34] were negatively associated with PA with a limited suggestive level of evidence. Even though mainly based on cross-sectional studies, the negative associations of ‘composite sedentary behavior’, ‘internet use’, and ‘reading’ are corroborated by the results of the MA, showing small to moderate negative effect sizes [29]. Regarding ‘vigorous PA’, Bruner and colleagues [39] suggested that a decline in PA over the course of the school year, in adolescents, is mainly due to a decrease in level of vigorous intensity activity rather than moderate intensity activity. Despite a clear dose-response specificity of low, moderate, and vigorous PA intensities on cardiovascular risk, metabolic health, osteoporosis, immune function, and mental health [40], the relationship between intensity (but also frequency, duration, and mode of activity) and adherence of PA is controversial [35, 41]. To deepen our understanding of the quality of the PA and to provide sound guidelines for PA prescriptions, further research on this area is recommended to consider the intensity of PA on both absolute (e.g., oxygen uptake, oxygen uptake relative to body mass, kcal or kJ per minute, and METs) and relative (e.g., percentages of maximal oxygen uptake, oxygen uptake reserve, heart rate reserve, maximal heart rate, Borg’s Rating of Perceived Exertion) terms [42].

The investigation of determinants in adults showed a higher proportion of negative associations (*n* = 6) with respect to positive associations (*n* = 1). Regarding the negative associations, a probable level of evidence was found only for ‘transition to university’ and ‘pregnancy/having a child’ with respect to overall PA/Ex in all ages >18 years, with the review showing a moderate quality [12]. Furthermore, ‘moving into an institution’ [12], ‘time limitation’ [24, 31], ‘language difficulties’ [24], and ‘smoking’ [27] were negatively associated but with a limited suggestive level of evidence. Conversely, ‘baseline activity level’ was positively associated with overall PA/Ex in adults ≥40 years but with a limited suggestive level of evidence and the quality of the review was moderate [27]. Although without a consistent convincing evidence among all the determinants, these findings highlight the possible impact of specific life events (i.e., ‘transition to university’, ‘pregnancy/having a child’, and ‘moving into an institution’) on the relationship between behavioral determinants and PA and the need for effective interventions and planning to increase health-enhancing PA.

An interactionist approach may help further our understanding of the role played by specific life events. Different life events with shorter-term (e.g., pregnancy) or longer-term effects (e.g., retirement) might exert their effects on PA behaviors separately, in different life phases. Differently, life events, whose shorter- and longer-lasting effects partially overlap in time (e.g., pregnancy and marital status) might generate interactive effects. Moreover, the impact of life events on PA behaviors might be moderated by other behavioral factors. For instance, the extent to which ‘retirement’ impacts actual PA behavior may be moderated by past PA habits. Moreover, the relationship linking the influence of different behavioral determinants of PA may be mediational in nature, with ‘time limitation’ probably mediating most of the life event effects on PA behaviors.

However, not only people older than 18 years should represent a target group population for the investigation of those life events and for considering health promotion interventions. Adequate attention should be placed also to those related to ‘job’, such as temporary or long-term unemployment, retirement, change in employment status, as well as to those concerning the ‘marital status’, such as starting a new close personal relationship, starting to live with someone, marriage, separation, divorce, widowhood, interpersonal loss, which are frequent during adulthood and may interfere and influence PA behaviors.

This umbrella SLR demonstrated that some potential determinants were found to be specific for each age group, whilst other determinants can be considered across all ages. For the latter, ‘screen use’ and ‘smoking’ were the most often investigated determinants, with six and four reviews examining the associations with several forms of PA, respectively. Actually, ‘screen use’ comprises several determinants, such as ‘TV viewing’, ‘TV viewing/playing video games’, ‘television viewing/sedentary’, ‘enjoy television viewing’, ‘video/computer game use’, ‘computer’, ‘screen time’, ‘video games’, which have been investigated for their potential associations with PA from preschool children to adolescents. The negative association between ‘enjoy television viewing’ and MVPA in preschool children emphasized the need for intervention programs to decrease screen related behaviors in the early years age group [26]. A similar negative association was also found in children with respect to after-school time periods (i.e., after-school PA) [30]. Particularly due to the fact that ‘TV viewing’ may negatively influence PA during time spent at home with high TV accessibility, intervention programs should be encouraged for increasing after-school PA [30]. Future research should adopt longitudinal study designs to investigate the effectiveness of long-term PA promoting strategies in children after school for several components, like fitness, health, cognitive functioning, engagement, motivation, psychological well-being, and social inclusion [43].

No consistent and conclusive association was found between ‘screen use’ and overall PA when children and adolescents were considered together [14, 26, 29, 32, 35]. However, a MA reported small to medium effect sizes between ‘TV viewing’ and PA and between ‘video/computer game use’ and PA and also considering possible moderators affecting the strength of their relationship [14]. In particular, the moderator analysis showed that only vigorous PA, but not combined, MVPA, and sports, is negatively associated with ‘TV viewing’. These and the present findings corroborate the need of further research on the effects of different variables of PA (e.g., intensity, duration, frequency) on PA behaviors. Moreover, the potential impact of the new technologies on PA behaviors of young people should be considered. Although TV viewing remains the most prominent leisure time sedentary behavior amongst youth, new techniques providing unlimited access to social and internet platforms (e.g., mobile phones and tablet computers) may have both a social and educational role for children and adolescents [29]. Recently, technologically augmented realities have been created to combine real and virtual objects in a real environment [44], also linked to PA (e.g., the smart phone game Pokémon Go). Such advancements in technology-driven PA (“exergaming”) [45, 46] are attracting the attention of decision makers involved in PA promotion as a potential innovative strategy for reducing physically inactive time, increasing adherence to exercise programs, and promoting enjoyment of PA [47]. However, this still very young line of research does not allow to draw definitive conclusions on effectiveness and long-term effects of exergaming and its role as a determinant of PA behaviors for different populations [48, 49].

Smoking is considered one of the biggest public health threats, killing around 6 million people globally every year [50]. It is associated with several diseases, including various types of cancer, coronary heart diseases, stroke, and chronic obstructive pulmonary diseases [50]. However, only one review with one primary study [27] reported a negative association between ‘smoking’ and overall PA/Ex in older adults (≥70 years old). Nevertheless, due to both the low quality of the primary study and its study design, Koeneman and colleagues [27] concluded to have insufficient evidence. Similarly, inconclusive associations were found for children and adolescents. Thus, they recommend additional research and the development and use of valid and reliable measurement instruments for both determinants and PA or Ex outcomes [27]. For the other reviews, the amount of primary studies providing negative associations did not reach 75% of the total amount of eligible studies. In particular, Kaczynski et al. [13] deeply examined the relationships between ‘smoking’ and overall PA and they found only 47% and 61% of the total studies included to report negative associations in youth and adult populations, respectively. Several factors need to be considered to explain these findings, such as the variety of definitions used to define a smoker and the differing terminology used to describe PA intensity [13]. Moreover, the use of PA as a harm-reduction strategy for smokers, the need of a threshold level of exercise to be reached for changes to occur, and the increased opportunities for smoking initiation due to the presence of peers for adolescents should also be considered. However, empirical evaluation of these explanations is necessary in order to clarify the possible relationship between ‘smoking’ and PA, which, at present, remains unclear [13].

Some determinants have received little attention so far and some new potential determinants need to be considered across the life course. In particular, future investigations on determinants of PA should study long time frames (e.g., from childhood to adulthood), to verify whether they predict active lifestyles with advancing years [37]. Among other determinants, life events require a high concern in their potential interactive or mediated effects on PA behaviors, starting from young populations. Our understanding of PA determinants could also benefit from investigations on sport participation at both young ages and during transition to adulthood, as well as on some dietary habits like alcohol consumption, which deserve to be better explored across the life course. In particular, it is surprising that alcohol consumption has been reported only in one SLR related to children. Finally, it could be advisable to examine how specific determinants of PA behaviors (e.g., ‘participation in organized sports’, ‘participation in sport teams outside school’, ‘sports activities’, ‘lifelong participation in PA’, ‘vigorous PA’, ‘previous PA’, ‘PE/school sports’) cluster with those related to unhealthy behaviors (e.g., ‘smoking’, ‘alcohol consumption’, ‘sedentary activities’, and screen use’).

Despite scholars from different disciplines actually contributed to the considerable amount of scientific papers, some methodological caveats have to be considered when analyzing and interpreting findings to guarantee core strength for the cumulated knowledge on determinants of PA behaviors. The first concern relates to the need of clear definitions of PA behaviors to prevent confusion and difficulty in interpretation of homonymous terms [42, 51]. To note, the present study was based on the consensus of the DEDIPAC-KH research team (consisting of 23 participants from five partner Nations) on a common nomenclature for PA that encompasses any bodily movement produced by skeletal muscles that results in energy expenditure, which may be unstructured and everyday life activity, exercise that includes prearranged, deliberate, and repetitive activity, and grassroots sports and competitive sports [36]. Despite this comprehensive definition ensures an extensive perspective of determinants of PA behaviors, it does not guarantee that the authors of the considered SLRs and MAs adopted the same pragmatic approach. The second concern pertains the absence of clear and well-established definitions used by scholars to summarize the information of determinants included in the primary studies they analyzed. To avoid misinterpretation of labels due to cultural biases, in the present umbrella SLR it was decided to refer to the actual terminology provided in the SLRs and MAs. The third concern refers to the difficulty in detecting information related to specific aspects of PA engagement, such as frequency and duration, and typology of exercise. Finally, a wide range of study designs, measurement techniques, population groups, determinants investigated, and PA outcomes emerged from the primary studies, making it difficult to evaluate all the evidence and to draw definitive conclusions. In particular, the most common study design used was cross-sectional, which limited the strength of any evidence. In fact only few determinants received a probable level of evidence. Moreover, the majority of PA outcomes were obtained by non-objective measurement methods, which provide less accurate data with respect to objective methods of PA evaluation.

## Conclusions

The promotion of PA should be pursued also considering the behavioral determinants. For youth, the most relevant evidence obtained by this umbrella SLR revealed the importance of being physically active in the early years, the necessity to increase active transportation to/from school, independent mobility, and the importance of being involved in ‘free-range activities’ that are away from adult supervision. Conversely, a reduction of time spent in front of screens, which is thought to negatively influence PA behaviors, needs to be carefully considered in light of the probable impact these new technologies have and the potential social and educational roles they play in the lives of young people. For adult populations, PA behaviors are mostly influenced by the life events, which represent a time constraint for possible involvement in PA.

The outcomes of the present umbrella SLR are not only limited to the identification of the behavioral PA determinants, which have a sufficient evidence-base to inform policy development, and of those determinants, which received little or no attention and are therefore in need for further research. The present findings also allow to suggest what type of research designs may be most useful for future research to yield stronger conclusions and generate substantial recommendations to multiple experts converging on the promotion of active lifestyles. Firstly, long-term prospective and longitudinal experimental designs should be employed to tackle the change in relevance of different determinants in critical transition phases from childhood to adulthood and from adulthood to older years. Secondly, an interactionist approach may help understand the joint contribution of different behavioral factors in determining PA behaviors and therefore the need to consider them jointly for effective PA promotion actions. Also, a mediational approach may allow to understand whether different behavioral determinants are directly liked to PA, or indirectly through a mediational chain, in order to identify which is the factor to be primarily targeted to obtain a positive PA behavior change. Finally, future research should address potential clustering and interactions of behavioral and non-behavioral (e.g., psychological, social, environmental) determinants, which may be crucial to inform multisectoral strategies of PA promotion.

## Abbreviations

Ce: Convincing evidence
DEDIPAC-KH: DEterminants of DIet and Physical ACtivity-Knowledge Hub
EU: European Union
EU-PAD: EUropean-Physical Activity Determinants
Ex: Exercise
Lns: Limited, no conclusive evidence
Ls: Limited suggestive evidence
MA: Meta-analysis
MVPA: Moderate-to-vigorous physical activity
PA: Physical activity
Pe: Probable evidence
SLR: Systematic literature review
